# Feasibility of canakinumab withdrawal in colchicine-resistant familial Mediterranean fever

**DOI:** 10.1093/rheumatology/kead128

**Published:** 2023-03-24

**Authors:** Seher Sener, Veysel Cam, Ezgi Deniz Batu, Muserref Kasap Cuceoglu, Zeynep Balik, Emil Aliyev, Yagmur Bayindir, Ozge Basaran, Yelda Bilginer, Seza Ozen

**Affiliations:** Division of Pediatric Rheumatology, Department of Pediatrics, Hacettepe University, Ankara, Turkey; Division of Pediatric Rheumatology, Department of Pediatrics, Hacettepe University, Ankara, Turkey; Division of Pediatric Rheumatology, Department of Pediatrics, Hacettepe University, Ankara, Turkey; Division of Pediatric Rheumatology, Department of Pediatrics, Hacettepe University, Ankara, Turkey; Division of Pediatric Rheumatology, Department of Pediatrics, Hacettepe University, Ankara, Turkey; Division of Pediatric Rheumatology, Department of Pediatrics, Hacettepe University, Ankara, Turkey; Division of Pediatric Rheumatology, Department of Pediatrics, Hacettepe University, Ankara, Turkey; Division of Pediatric Rheumatology, Department of Pediatrics, Hacettepe University, Ankara, Turkey; Division of Pediatric Rheumatology, Department of Pediatrics, Hacettepe University, Ankara, Turkey; Division of Pediatric Rheumatology, Department of Pediatrics, Hacettepe University, Ankara, Turkey

**Keywords:** canakinumab, colchicine resistance, FMF

## Abstract

**Objectives:**

There is no consensus on canakinumab treatment tapering and discontinuation strategies in colchicine-resistant FMF patients. In this study, we aimed to establish a treatment management and discontinuation protocol in paediatric FMF patients treated with canakinumab.

**Methods:**

Fifty-eight FMF patients treated with canakinumab were included. Since 2020, we have applied a protocol based on our experience whereby canakinumab is administered monthly in the first 6 months, followed by bimonthly for 6 months, and a final period of every 3 months (for 6 months). The patients were divided into two groups: 2012–2019 (group A) and 2020–2022 (group B).

**Results:**

In group A (*n* = 33), the median duration of canakinumab treatment was 2.5 years [interquartile range (IQR) 1.9–3.7]. A total of 25 of 33 patients discontinued canakinumab after a median of 2.1 years (IQR 1.8–3.4). In two patients, canakinumab was restarted because of relapse. In group B (*n* = 25), canakinumab was discontinued in 18 patients at the end of 18 months. After a median follow-up of 0.8 years (IQR 0.6–1.1), two patients had a relapse and canakinumab treatment was reinitiated. The remaining 16 patients still have clinically inactive disease and are receiving only colchicine. When we compared the characteristics between groups A and B, there were no significant differences regarding demographics, clinical features, and outcomes.

**Conclusion:**

This is the largest study in the literature suggesting a protocol for discontinuing canakinumab in paediatric FMF patients. It was possible to discontinue canakinumab successfully in more than half of the patients in 18 months. Thus we suggest that this protocol can be used in paediatric FMF patients.

Rheumatology key messages

- There is no optimal treatment protocol for reducing and discontinuing canakinumab in paediatric FMF patients.
- We created a protocol for discontinuing canakinumab in FMF based on our clinical experience.
- With this protocol, canakinumab was successfully discontinued in 64% of FMF patients after 18 months.

## Introduction

FMF is very common in the eastern Mediterranean and is characterized by recurrent, self-limiting episodes of fever and serositis [1, 2]. The primary aim of FMF treatment is to treat subclinical inflammation and reduce the frequency of clinical attacks [3]. Inadequate treatment may result in complications such as secondary amyloidosis, which is associated with severe morbidity and mortality [4, 5].

FMF is caused by gain-of-function mutations of the *MEFV* gene, which encodes pyrin, a protein that exerts a suppressive effect on inflammasome activation [5, 6]. This inhibitory control mechanism is lost as a result of mutations in the *MEFV* gene. Inflammasome activation leads to the conversion of procaspase 1 into caspase 1 and subsequent cleavage of pro-IL-1β into mature IL-1β, which is secreted from cells [6]. Inflammation resulting from this overactivation of pyrin inflammasomes drives the typical febrile inflammatory attacks observed in FMF [7].

Colchicine, the first-line treatment of FMF, is quite effective in controlling both attacks and preventing the development of amyloidosis [8]. Nevertheless, unresponsiveness or resistance to colchicine occurs in ≈5–10% of patients [9]. In addition, colchicine cannot be used at effective doses in some patients due to its side effects or intolerance [10]. Therapeutic blockage of IL-1β is highly effective in the treatment of FMF patients with colchicine resistance or intolerance [11, 12]. Among the anti-IL-1 agents, anakinra (recombinant antagonist of the IL-1 receptor), canakinumab (human monoclonal antibody against IL-1β) and rilonacept (a soluble decoy receptor ‘trap’, binding both IL-1α and IL-1β) can be used in the treatment of FMF [13]. The CLUSTER study (NCT02059291) has shown canakinumab to be effective and safe in colchicine-resistant FMF patients [14].

There is no consensus in the literature on the optimal treatment duration and protocol of tapering and discontinuation of canakinumab in paediatric FMF patients. Moreover, studies on this subject are very limited. In this study, we share our 10-year experience of treatment with canakinumab in colchicine-resistant FMF patients and we suggest a protocol for treatment monitoring and discontinuing the drug.

## Patients and methods

Children (0–18 years) with FMF treated with canakinumab who were followed between January 2012 and December 2022 at the Pediatric Rheumatology Unit of Hacettepe University Faculty of Medicine, Ankara, Turkey, were included in the study. All included patients met the Eurofever/Paediatric Rheumatology International Trials Organisation criteria for FMF [15]. Colchicine resistance was defined as continued disease activity [as reflected by either recurrent clinical attacks (average one or more attacks per month over 3 months) or persistently elevated acute phase reactants between attacks] in a patient despite the maximum tolerated dose of colchicine [16]. Clinically inactive disease was defined as no active clinical symptoms, normal acute phase reactants, and normal disease activity scores. Complete remission on canakinumab was defined as an inactive disease for at least 6 months while treatment with canakinumab was ongoing. Complete remission off canakinumab was defined as clinical remission for at least 6 months without the use of canakinumab.

All FMF patients were on colchicine treatment, as well. All received anakinra (2 mg/kg/dose, maximum 100 mg/day) before canakinumab (2 mg/kg/dose maximum 150 mg/dose) since it is required to start the biologic treatment first with anakinra, according to the rules of the Social Security Institution Health Practice Communiqué in our country. Demographics and clinical findings, laboratory results, treatments and outcomes of the patients at diagnosis were reviewed retrospectively. *MEFV* gene variant analysis was performed with Sanger sequencing. Twelve variants (*E148Q*, *P369S*, *F479I*, *M680I*, *M680I*, *I692del*, *M694V*, *M694I*, *K695R*, *V726A*, *A744S*, and *R761H*) were tested in the *MEFV* gene in our Department of Medical Biology.

The disease activity was evaluated using a patient/parent visual analogue scale (VAS) (0–10 cm), physician VAS (0–10 cm) and Auto-Inflammatory Diseases Activity Index (AIDAI) score (active disease >9 points) in addition to acute phase reactants (ESR and high-sensitivity CRP) [17, 18].

There was no standard protocol for canakinumab treatment before 2020 and patients were treated according to the judgement of the physician. The treatment target was complete remission on medication. With our experience in these 10 years, we implemented a standard protocol for treatment reduction and discontinuation starting in 2020. We divided patients into two groups: patients who received canakinumab treatment before 2020 (group A) and those who received canakinumab treatment between 2020 and 2022 (group B). All patients in group B were started on canakinumab treatment between January 2020 and December 2020. Canakinumab treatment was discontinued in June 2022 in the last patient whose treatment was discontinued.

### Treatment reduction and discontinuation strategy for canakinumab

**Figure 1.**
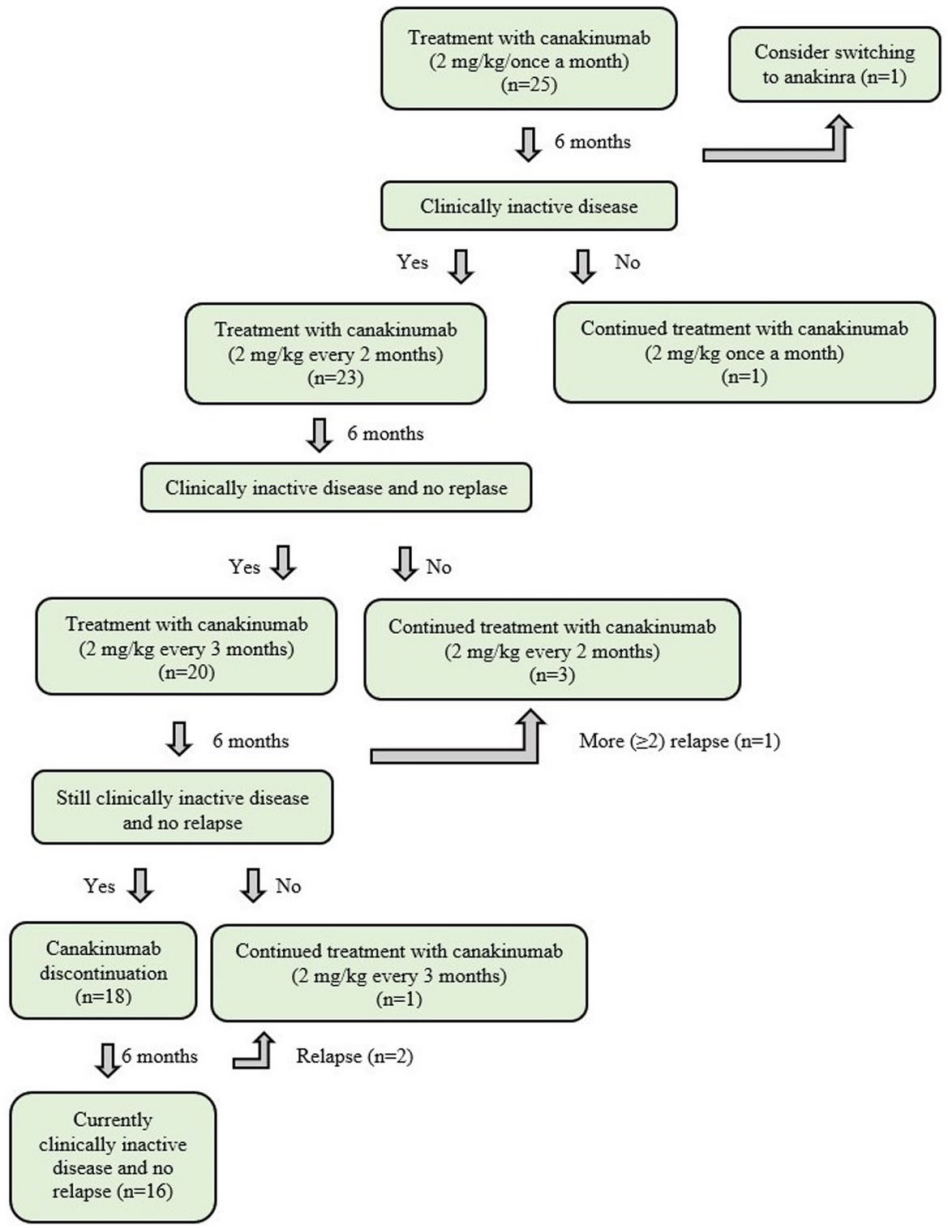
Schematic diagram of the canakinumab discontinuation protocol in group B

Our canakinumab protocol is presented in Fig. 1. Canakinumab treatment is administered every month for 6 months; the dosing interval is extended to 2 months in clinically inactive patients. At month 12, 2-month intervals are extended further to 3 months if the patient remains clinically inactive. Then two more doses of canakinumab are administered 3 months apart. Treatment of patients who are still clinically in remission is terminated at 18 months. If clinically inactive disease is not achieved, dose intervals are not extended. If the disease becomes active after the extension of the dose intervals, the dosing interval is immediately changed to the interval in the previous step. During this period, the patients who could not tolerate the 3-month dose intervals were returned to the treatment regimen with 2-month intervals.

Clinical findings, acute phase reactants, patient/parent and physician VASs and AIDAI parameters of all patients before starting anti-IL-1 therapy (day 0), at 1, 3, 6, 9, 12, 15 and 18 months after starting canakinumab therapy and after discontinuation of canakinumab therapy are evaluated. In addition, patients are also monitored for adverse events or side effects such as infection and local skin reactions related to canakinumab.

### Statistical analysis

Statistical analyses were performed using SPSS version 24 (IBM, Armonk, NY, USA). The variables were investigated using visual and analytic methods (Shapiro–Wilk test) to determine whether they were normally distributed. Descriptive statistics were presented as medians (IQRs) for continuous variables and as numbers and percentages for nominal/categorical variables. Differences in proportions between groups were evaluated by the chi-squared test or Fisher’s exact and Mann–Whitney *U* test where applicable. *P*-values <0.05 were considered statistically significant.

The study was approved by the ethics committee of our hospital (GO 19/635). Written consent from the patients/parents was obtained according to the Declaration of Helsinki (1964). In addition, permission for the off-label use of both anakinra and canakinumab was granted by the Turkish Ministry of Health for each patient.

## Results

### Characteristics of the patients

A total of 58 paediatric FMF patients (58.6% female) were included in the study group. The median ages of the patients at diagnosis and the first biologic treatment were 4.1 years (IQR 2.3–5.9) and 13.6 years (IQR 6.3–16), respectively. The demographic and clinical features of the patients are summarized in Table 1.

**Table 1.** Characteristics of FMF patients treated with canakinumab

Characteristics	All patients (*N* = 58)	Group A (*n* = 33)	Group B (*n* = 25)	*P*-value
Female, *n* (%)	34 (58.6)	19 (57.6)	15 (60)	0.853
Age at symptom onset, years	2.5 (1.1–3.7)	2.1 (0.9–3.3)	2.5 (1–3.8)	0.626
Age at diagnosis, years	4.1 (2.3–5.9)	4.1 (2.1–5.5)	4.2 (1.5–6.8)	0.777
Age at first biologic treatment, years	13.6 (6.3–16)	12.7 (5.8–14.7)	14.2 (6.9–15)	0.233
Time between symptom onset and diagnosis, years	1.3 (0.9–3.1)	1.5 (1.1–3.3)	1.1 (0.8–3)	0.300
Time between diagnosis and biologic treatment, years	5.6 (4.1–11.3)	5.6 (3.9–12)	7.7 (5.2–13.1)	0.233
Time between anakinra and canakinumab treatment, months	3 (2–3)	3 (2–3)	3 (1–3)	0.519
Parental consanguinity, *n* (%)	16 (22.2)	5 (15.2)	9 (36)	0.066
Comorbidity, *n* (%)	19 (32.8)	12 (36.4)	7 (28)	0.564
Clinical features before biologic treatment, *n* (%)				
Fever	56 (96.5)	32 (97)	24 (96)	1.000
Abdominal pain	47 (81.1)	25 (75.8)	22 (88)	0.320
Arthritis	30 (51.7)	12 (36.4)	18 (72)	0.007
Chest pain	23 (39.7)	10 (30.3)	13 (52)	0.094
Erysipelas-like erythema	13 (22.4)	3 (9.1)	10 (40)	0.005
*MEFV* mutations, *n* (%)				
* M694V*/*M694V*	42 (72.4)	20 (60.6)	22 (88)	0.021
* M694V*/*M680I*	9 (15.5)	7 (21.2)	2 (8)	0.169
* M694V*/*V726A*	3 (5.2)	2 (6.1)	1 (4)	0.104
* M680I*/*M680I*	2 (3.4)	2 (6.1)	0	–
* M680I*/*V726A*	1 (1.7)	1 (3)	0	–
* M694V*/*M694I*	1 (1.7)	1 (3)	0	–
Number of attacks in the year before biologic treatment	12 (9–12)	12 (9–12)	12 (8–14)	0.550
Laboratory findings before biologic treatment				
ESR, mm/h (0–20)	31 (23–62)	35 (25–66)	32 (25–63)	0.239
CRP, mg/dL (0–0.5)	4.8 (4.5–9)	4.5 (4.3–9.2)	4.7 (4.6–8.9)	0.289
Patient/parent VAS before biologic treatment (0–10 cm)	7 (6–9)	8 (4–9)	8 (6–10)	0.138
Physician VAS before biologic treatment (0–10 cm)	6 (5–7)	6 (4–8)	7 (3–8)	0.243
AIDAI score in the month before biologic treatment	11 (9–15)	12 (9–16)	11 (7–13)	0.417
Number of attacks in the year after biologic treatment	0 (0–1)	0 (0–1)	0 (0–1)	0.278
Laboratory findings after biologic treatment				
ESR, mm/h (0–20)	6 (3–14)	7 (3–16)	5 (2–16)	0.281
CRP, mg/dL (0–0.5)	0.3 (0.1–1)	0.3 (0.1–0.9)	0.4 (0.1–1)	0.314
Patient/parent VAS after biologic treatment (0–10 cm)	0 (0–3)	0 (0–3)	0 (0–4)	1.000
Physician VAS after biologic treatment (0–10 cm)	0 (0–2)	0 (0–2)	0 (0–3)	1.000
AIDAI score in the month after biologic treatment	0 (0–2)	0 (0–2)	0 (0–2)	1.000
Patients currently receiving canakinumab monthly, *n* (%)	1 (1.7)	0	1 (1.7)	–
Patients whose canakinumab dosing interval was extended to 2 months, *n* (%)	54 (93.1)	32 (97)	23 (92)	0.449
Patients whose canakinumab dosing interval was extended to 3 months, *n* (%)	36 (62.1)	16 (48.5)	20 (80)	0.014
Patients who successfully discontinued canakinumab therapy, *n* (%)	39 (67.9)	23 (69.7)	16 (64)	0.442
Total follow-up duration, years	10.9 (6.6–14.7)	10.9 (7.7–15)	11.8 (7.3–14.2)	0.626
Duration under canakinumab treatment, years	1.6 (1.5–2.8)	2.5 (1.9–3.7)	1.5 (1.5–1.6)	<0.001
Follow-up duration after discontinuation of canakinumab, years	1.2 (1.1–3.7)	2.7 (1.7–4.5)	0.8 (0.6–1.1)	<0.001
Relapse under canakinumab, *n* (%)	11 (18.9)	6 (18.2)	5 (20)	0.293
Relapse after discontinuation of canakinumab, *n* (%)	3 (5.2)	2 (6.1)	2 (8)	0.251
Outcome, *n* (%)				
Complete remission off canakinumab	39 (67.9)	23 (69.7)	16 (64)	0.442
Complete remission on canakinumab	9 (15.5)	5 (15.1)	4 (16)	0.340
Partial remission on canakinumab	7 (12.1)	4 (12.1)	4 (16)	0.089
Progression on canakinumab	3 (5.2)	1 (3)	1 (4)	1.000

Data are presented as median (IQR) unless stated otherwise.

All of our patients had ongoing disease activity despite receiving colchicine at the maximum tolerated dose, thus anti-IL-1 treatment was started. All had elevated acute phase reactants, high patient/parent and physician VAS values [median 7 (IQR 6–9) and 6 (5–7), respectively] and high AIDAI scores [median 11 (IQR 9–15)] before biologic treatment. A significant decrease was observed in acute inflammatory markers, VAS values (both patient/parent and physician) and AIDAI scores in all patients after starting biologic treatment (*P* < 0.001).

Between 2012 and 2020, 33 (56.9%) patients were treated with canakinumab without following a standard protocol (group A). Starting in 2020, 25 (43.1%) patients were treated according to the canakinumab treatment protocol we developed (group B).

In group A (*n* = 33), the median duration of canakinumab treatment was 2.5 years (IQR 1.9–3.7). Treatment was adjusted individually from patient to patient, with a target of complete remission. The canakinumab dose interval was extended to 2 months in 32 (97%) patients and 3 months in 16 (48.5%) patients in this group. In one patient (3%) whose dose interval could not be extended to 2 months, anakinra treatment was started again due to failure to respond to canakinumab. In four patients (12.1%), the dosing interval was extended to 2 months and stopped without extending it to 3 months. Eventually, 25 of 33 patients (75.7%) discontinued canakinumab after a median of 2.1 years (IQR 1.8–3.4). In two of these patients (8%), canakinumab was restarted because of relapse. The treatment could not be stopped in seven patients. Of these seven patients, one had secondary amyloidosis, four had an attack when the treatment interval was extended and complete remission could not be achieved in two.

In group B (*n* = 25), the median duration of canakinumab treatment was 1.5 years (IQR 1.5–1.6). According to our suggested protocol, canakinumab 2 mg/kg once a month was started in these patients for the first 6 months (Fig. 1). In the 23 patients (92%) with clinically inactive disease, the dosing interval was extended to 2 months after the first 6 months. In two patients, the dosing interval could not be extended to 2 months because the disease was not inactive under canakinumab treatment. In one of these patients, anakinra treatment was started due to failure to respond to canakinumab treatment. After the second 6 months, the dosing interval of canakinumab was extended to 3 months in 20 patients (80%) with clinically inactive disease. The dose interval remained at 2 months in three patients due to intermittent disease activations. At the end of the third 6-month period (after a total of 18 months and 11 doses of canakinumab treatment), canakinumab was discontinued in 18 patients (72%) who had clinically inactive disease at the final evaluation (Fig. 1). Canakinumab treatment was continued in two patients. Moreover, the dosing interval was changed to 2 months in one of these two patients because of frequent attacks. In the first 6-month follow-up of 18 patients whose canakinumab treatment was discontinued, canakinumab was started again in only two patients (8%) due to relapse. Canakinumab was re-administered to these patients at 3-month intervals. As a result, canakinumab was successfully discontinued in 16 of 25 patients (64%) after 18 months in this group. These patients still have clinically inactive disease and are receiving only colchicine therapy.

The demographics, clinical features [apart from arthritis (*P* = 0.007) and erysipelas-like erythema (*P* = 0.005)] and variables associated with disease activity were not significantly different between group A and group B patients (Table 1). The duration of canakinumab treatment was longer in group A than in group B patients [2.5 years (IQR 1.9–3.7) *vs.* 1.5 (1.5–1.6), *P* < 0.001]. However, there was no difference between relapse rates after discontinuation of canakinumab and complete remission off drug in patients treated with canakinumab in both groups A and B (*P* = 0.251 and *P* = 0.442, respectively). The minimum follow-up period for the patients in group B was 6 months. Having said that, it should be noted that the follow-up after canakinumab withdrawal was significantly shorter in group B compared with group A [0.8 years (IQR 0.6–1.1) *vs.* 2.7 (1.7–4.5), *P* < 0.001]. The canakinumab treatment durations, outcomes and follow-up periods after canakinumab discontinuation of the patients in group B are presented in Supplementary Table S1, available at *Rheumatology* online. When we compared the characteristics of patients who discontinued canakinumab successfully between group A and group B, there was no significant difference regarding demographics, clinical features and outcomes.

In the whole group, no serious side effects were observed during treatment with canakinumab. Only two patients (3.4%) had mild flu-like symptoms.

## Discussion

We suggest a protocol for tapering and discontinuing canakinumab in paediatric FMF patients. This protocol was developed based on our initial 10-year experience in treating colchicine-resistant FMF patients with canakinumab. In the last 2 years we have used our suggested protocol, achieving a similar success with shorter and less use of canakinumab . Furthermore, we were able to offer a fixed protocol for the patients. Our results showed that canakinumab could be successfully discontinued in more than half our patients (64%) in 18 months.

The efficacy and safety of canakinumab in patients with colchicine-resistant FMF have been demonstrated in the literature [14, 19–22]. However, we still lack data regarding the adequate treatment duration and interval information, where studies are quite limited. Akarcan *et al.* [23] presented their experience with canakinumab in nine paediatric FMF patients with colchicine resistance. Monthly canakinumab (a total of six doses) was administered to all patients as initial therapy during the first 6 months. For the next 6 months, canakinumab (three doses) was administered bimonthly (maintenance therapy). After a total of nine doses, canakinumab treatment was discontinued and the patients were followed up for attacks. Canakinumab was re-administered at 3-month intervals (continuation therapy) to patients who developed new attacks. Four of the patients developed an attack 9.0 months (s.d. 2.9, IQR 6–12) after discontinuation of treatment and switched to the continuation treatment period, with no more attacks. As a result of this study, they suggested that the protocol they created could be used successfully in colchicine-resistant paediatric FMF patients [23].

In a recent study by Jarabulus *et al.* [24] in adult FMF patients, 57 patients treated with canakinumab were evaluated retrospectively. All patients were started on canakinumab at a dose of 150 mg once a month and the dose and interval were not changed for the first 6 months. Although there was a decrease in the frequency and duration of attacks in 35 of 57 patients who received monthly treatment, intermittent increases in acute phase reactants were observed in the periods between attacks in 13 of them. Therefore, except for the 13 patients, the dosing interval of canakinumab was extended in the remaining 22 patients after the first 6 months and the treatment was completely discontinued in 12 of these patients who did not have an attack in the last 6 months. The dosing intervals for the remaining 10 patients were extended to 8–12 weeks after 6 months of canakinumab treatment. Three of the 12 patients whose treatment was discontinued started monthly treatment again after the attacks recurred. Nine patients are still being followed without attacks and are receiving only colchicine treatment [24]. Although the authors rightly argued that the course of FMF differs from person to person and treatment should be adjusted individually, there was no clear schedule for drug discontinuation in their study.

We did not have a standard protocol for the canakinumab dose range before 2020 (group A, *n* = 33). However, 25 patients (43.1%) were treated in accordance with the canakinumab treatment protocol we developed (after 2020, group B). Although the duration of canakinumab treatment was shorter in group B [1.5 years (IQR 1.5–1.6) *vs* 2.5 (1.9–3.7)], there was no statistically significant difference between acute phase reactants, VAS values (both patient/parent and physician) and AIDAI scores in the two groups. Another important point we want to emphasize is that there was no difference between the rates of relapse after discontinuation of canakinumab and complete remission off canakinumab in patients treated with canakinumab in both groups A and B (*P* = 0.251 and *P* = 0.442, respectively). This suggests that our canakinumab reduction and discontinuation protocol has been successful. However, patients in group B after discontinuation of canakinumab had a shorter follow-up [median 0.8 years (IQR 0.6–1.1)].

The most significant limitation of our study is the short follow-up, especially in group B patients whose canakinumab treatment was tailored according to the protocol we proposed. Longer follow-up results will be valuable to guide the management of these FMF patients. Our study also has the limitations inherent in retrospective studies. Another limitation is that there was no standard evaluation of compliance based on the retrospective character of the study, although, during the routine visits, we carefully asked for colchicine compliance of both patients and parents. Finally, we had serum amyloid A in a limited number of patients, as it is not in routine use in our centre.

## Conclusion

In this study we proposed an algorithm for tapering and discontinuing canakinumab in colchicine-resistant FMF patients. With this protocol it was possible to discontinue canakinumab successfully in 18 months in more than half of our patients. Our results need to be assessed with a longer follow-up and external validation of our protocol.

## Supplementary Material

kead128_Supplementary_Data

## Data Availability

The data are available from the corresponding author upon reasonable request.
