# Supplementary material for: Feasibility of canakinumab withdrawal in colchicine-resistant familial Mediterranean fever
Source: Rheumatology (Oxford). 2023 Mar 24;62(11):3700–5. doi: 10.1093/rheumatology/kead128 (PMC10629778; doi:10.1093/rheumatology/kead128)
Supplement: kead128_Supplementary_Data [file kead128_supplementary_data.docx]

**Supplementary Table S1.** Canakinumab treatment durations, outcomes, and follow-up periods after canakinumab discontinuation in Group B patients

| **Patients no.** | **Duration of canakinumab treatment, months** | **Follow-up after discontinuation of canakinumab, months** | **Outcome** |
| --- | --- | --- | --- |
| 1 | 18 | 16.5 | Complete remission “off canakinumab” |
| 2 | 18 | 15.5 | Complete remission “off canakinumab” |
| 3 | 18 | 14 | Complete remission “off canakinumab” |
| 4 | 3 | 28 | Treatment switch to anakinra |
| 5 | 18 | 13.5 | Complete remission “off canakinumab” |
| 6 | 33 | - | Continued treatment with canakinumab (every 2 months) |
| 7 | 18 | 12.5 | Complete remission “off canakinumab” |
| 8 | 18 | 12.3 | Complete remission “off canakinumab” |
| 9 | 18 | 12 | Complete remission “off canakinumab” |
| 10 | 28 | - | Continued treatment with canakinumab (every 2 months) |
| 11 | 18 | 10 | Complete remission “off canakinumab” |
| 12 | 18 | 9 | Complete remission “off canakinumab” |
| 13 | 18 | 9 | Complete remission “off canakinumab” |
| 14 | 27 | - | Continued treatment with canakinumab (every 3 months) |
| 15 | 18 | 8.5 | Complete remission “off canakinumab” |
| 16 | 18 | 8.5 | Complete remission “off canakinumab” |
| 17 | 26 | - | Continued treatment with canakinumab (every 3 months) |
| 18 | 25 | - | Continued treatment with canakinumab (every 2 months) |
| 19 | 18 | 7 | Complete remission “off canakinumab” |
| 20 | 18 | 7 | Complete remission “off canakinumab” |
| 21 | 25 | - | Continued treatment with canakinumab (once a month) |
| 22 | 18 | 6.5 | Complete remission “off canakinumab” |
| 23 | 18 | 6 | Complete remission “off canakinumab” |
| 24 | 24 | - | Continued treatment with canakinumab (every 3 months) |
| 25 | 21 | - | Continued treatment with canakinumab (every 2 months) |
